# Large positive magnetoresistance in semiconducting single-walled carbon nanotubes at room temperature†

**DOI:** 10.1039/c8ra00877a

**Published:** 2018-03-13

**Authors:** Jean Pierre Nshimiyimana, Jian Zhang, Xiannian Chi, Xiao Hu, Pei Wu, Siyu Liu, Jia Liu, Weiguo Chu, Lianfeng Sun

**Affiliations:** CAS Key Laboratory of Nanosystem and Hierarchical Fabrication, CAS Center for Excellence in Nanoscience, National Centre for Nanoscience and Technology Beijing 100190 China slf@nanoctr.cn wgchu@nanoctr.cn; University of Chinese Academy of Sciences Beijing 100049 China

## Abstract

We have investigated the effect of a magnetic field on the resistance (magnetoresistance, MR) of single-walled carbon nanotube (SWNT) arrays. The SWNT devices consist of a mixture of metallic and semiconducting SWNTs between palladium electrodes. The MR of the devices is studied at room temperature and in the presence of perpendicular magnetic fields up to 0.24 tesla. The resistance increases as the external magnetic field becomes higher, suggesting a positive MR of SWNTs. After etching the metallic SWNTs by electrical breakdown, the MR can be further enhanced. Large positive MR values about 15%, 20% and 25% were found in three different devices at 0.24 tesla for semiconducting SWNTs at room temperature. Our results show potential for the development of magneto-electronic devices that are operable at room temperature.

## Abstract 2

A positive magnetoresistance is reported in single-walled carbon nanotubes at room temperature, which is further enhanced by etching metallic nanotubes.

## Graphical abstract

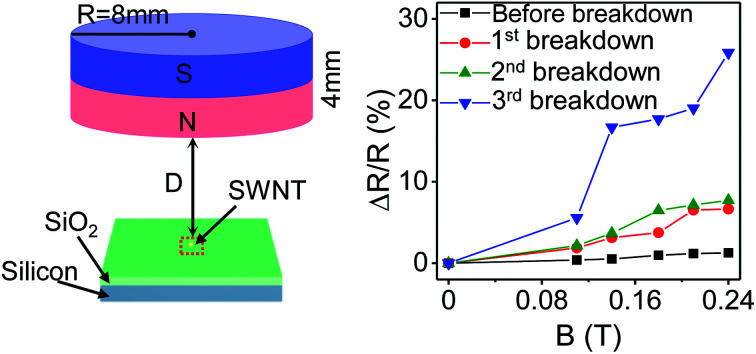

## Introduction

Owing to their unique and outstanding mechanical and electronic properties, single-walled carbon nanotubes (SWNTs) have shown great promise for applications in nanoscience and technology.^1^ These long, thin, cylindrical carbon molecules exhibit either metallic or semiconducting behaviour, which makes them a perfect candidate for next generation electronic devices, such as transistors, sensors and memory devices.^2–6^ Consequently, in order to evaluate their full potential in these devices, it is essential to not only understand but also to be able to control their electronic behaviour. Studies have shown that the electronic properties of SWNTs can be tailored by the application of external magnetic fields, resulting in strong conductance modulations.^7,8^ This effect called magnetoresistance (MR) is defined as the change of material's resistivity when it is placed in an external magnetic field. The study of MR effect has been of substantial importance for the development of a new class of devices, spin-electronic devices,^9^ in which both the charge and spin of the electron are utilized to store and process information.

For large-scale electronic applications, devices made of SWNTs arrays offer many advantages over individual SWNTs devices, due to their increased output currents and improved uniformity and reproducibility.^10,11^ As such, numerous studies involving the MR measurements in SWNTs arrays have been reported.^12–15^ In these systems, at temperatures below 200 K, a negative MR was observed at low magnetic fields followed by the saturation of the resistance at high magnetic fields and a possible upturn at lower temperatures with a quadratic field dependent positive MR. The negative MR was attributed to weak localization effect while a positive MR was considered due to strong localization of electrons, described within the framework of variable range hopping mechanism. However, while high values of MR are found at low temperatures and high magnetic fields, actual devices must practically operate at room temperature and in low magnetic fields.

Here we report the room temperature MR properties of SWNT arrays in magnetic fields from 0 up to 0.24 tesla. In a sample containing a mixture of both semiconducting and metallic SWNTs, a positive MR was observed. The MR was found independent on the length of the SWNTs between the two electrodes and largely dependent on the metallic and semiconducting nature of SWNTs.

## Experimental

**Fig. 1:**
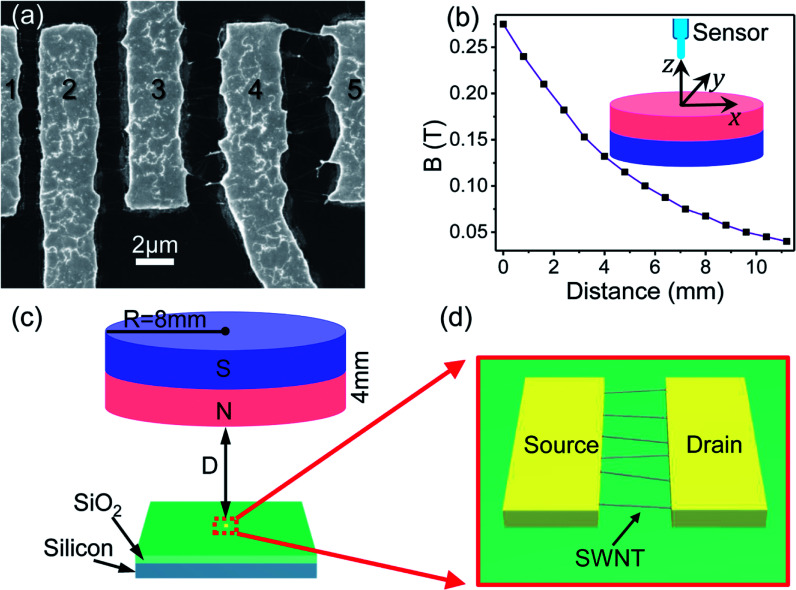
(a) SEM image showing different devices consisting of SWNT arrays between a pair of electrodes labeled as 1, 2, 3, 4 and 5. The spacing between electrodes 1–2, 2–3, 3–4 and 4–5 is 1 μm, 2 μm, 3 μm and 4 μm, respectively. (b) The magnitude of the magnetic field produced by a magnet along the *z*-direction. The inset is a schematic diagram showing the magnet and how to measure the magnetic field along the *z*-direction. The origin of the coordinates is at the center of the top surface of the magnet. The first data point is measured at *z* = 0. (c) Schematic diagram showing the magnetoresistance measurements setup. The SWNT device is placed below the center of the magnet at a distance *D* with coordinates (0, 0, *z*). The distance *D* has typical values of several millimeters and the length of SWNTs is about 1–4 micrometers. (d) Schematic diagram of the SWNT array device.

The SWNT devices used in this work were fabricated following a technique reported recently.^16^ Silicon wafer coated with 800 nm thick SiO_2_ layer was used as a substrate. Palladium electrodes were defined by electron beam lithography and deposited on the substrate by thermal evaporation and lift-off process. A 400 nm thick silver layer was evaporated on top of the substrate. SWNT were grown between electrodes by floating catalytic chemical vapour deposition^17^ in a mixture of 1000 sccm argon and 10 sccm methane at 1000 °C and deposited on the silver surface. The next and final stage consisted of thermally annealing the devices at 960 °C. At this temperature, the silver film melted into liquid, moved towards the electrodes and pulled the SWNTs on either side of the electrodes, which resulted in a straightened array of SWNTs between the two electrodes. The fabrication mechanisms are based on a dynamic motion of silver liquid to align SWNTs between prefabricated electrodes in high temperature annealing treatment.^16^ Transmission electron microscopy (TEM) and Raman spectroscopy have been used to characterize the SWNTs and they are shown in ESI as Fig. S1.† From the TEM image, the SWNTs are identified with diameter ∼1.5 nm. Moreover, the characteristic peaks (such as radial breathing mode (RBM), D-peak and G-peak) of SWNTs Raman spectroscopy are observed, which also can exclude the possibility of a carbon–silver mixture. Fig. 1a shows a scanning electron microscopy (SEM) image of the SWNT array devices. We fabricated various devices with different spacing between electrodes (1 μm, 2 μm, 3 μm and 4 μm). The SEM result showed that the alignment of SWNTs depends on the spacing between electrodes and the device with small spacing has the best alignment.

The MR measurements were done at room temperature using B1500 Agilent semiconductor characterization system and in the presence of a magnetic field produced by a permanent magnet. At first, we measured the magnetic field of the magnet along the *z*-direction. The inset of Fig. 1b displays the schematic diagram of the magnet and how the magnetic field was measured. In our experiment, a cylindrical permanent magnet (NdFeB) was used, whose radius and thickness are 8 mm and 4 mm, respectively. The magnetic field of the magnet was measured by means of a magnetic sensor (GM55 digital gaussmeter). The probe of the sensor was placed in the centre of the magnet to measure the first data point. Then the probe was moved along the *z*-direction and different data points were measured as shown in Fig. 1b.

The MR experiment was performed by placing the SWNT device below the centre of the magnet at a distance *D* with coordinates (0, 0, *z*). The magnetic field is always perpendicular to the SWNTs as shown in Fig. 1c. The distance *D* has typical values of several millimeters. Fig. 1d shows the schematic diagram of the SWNT device between the electrodes. The width of the electrodes is about 4 μm and the length of SWNTs is about 1–4 μm. Since the size of the magnet and its distance from the device are much larger than the length of SWNTs, the magnetic field at the SWNTs is vertical and the effect of fringe fields can be neglected. By measuring the distance *D* between the magnet and the device, the corresponding values of the applied magnetic field could then be obtained.

## Results and discussion

**Fig. 2:**
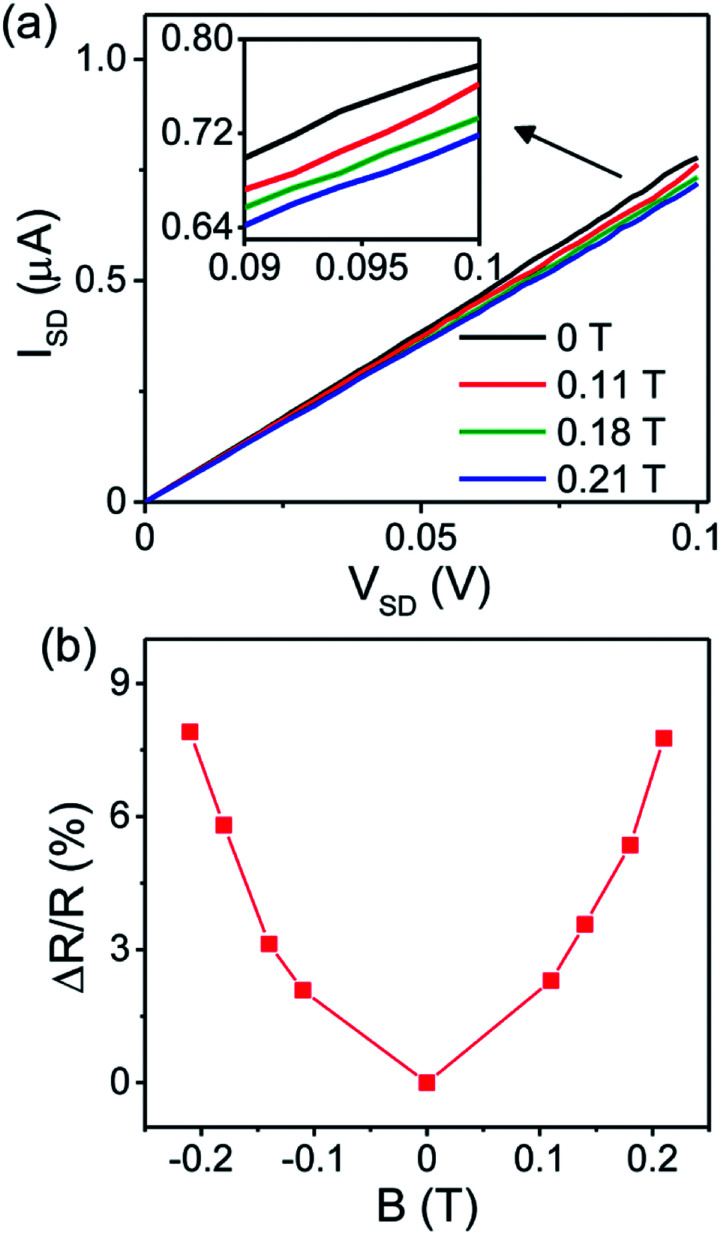
(a) Source-drain current (*I*_SD_) *versus* source-drain voltage (*V*_SD_) characteristics of the SWNT device under different magnetic fields at room temperature. (b) Magnetic field dependence of the magnetoresistance in both positive and negative magnetic fields. Symbols represent experimental data and solid lines are a guide to the eye.

Fig. 2a demonstrates source-drain current–voltage (*I*_SD_–*V*_SD_) characteristic curves of a SWNT device measured at room temperature under various magnetic fields from 0 up to 0.21 tesla. It can be seen that the application of the magnetic field suppresses the current and hence increases the resistance (*R* = *V*_SD_/*I*_SD_) of the device. An increase in the magnetic field further increases the resistance, indicating a positive MR whose ratio is defined as:
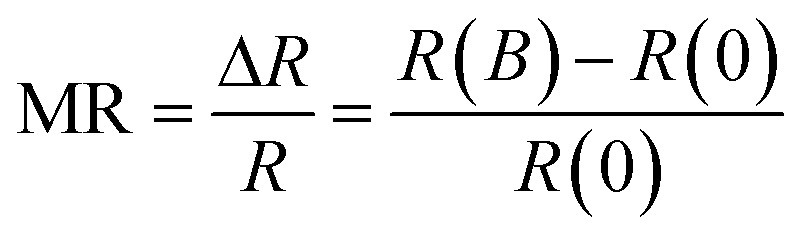
where *R*(*B*) is the electrical resistance at a magnetic field *B* and *R*(0) is the resistance at zero magnetic field. Fig. 2b shows the magnetic field dependence of the MR with different orientations of magnetic field. In this work, we define positive and negative magnetic field depending on its orientation with respect to the *z*-axis. It can be seen that the MR has the same behaviour and varies almost symmetric at positive and negative magnetic field, meaning that for the same absolute value of magnetic field, the magnitude of MR is almost the same.

**Fig. 3:**
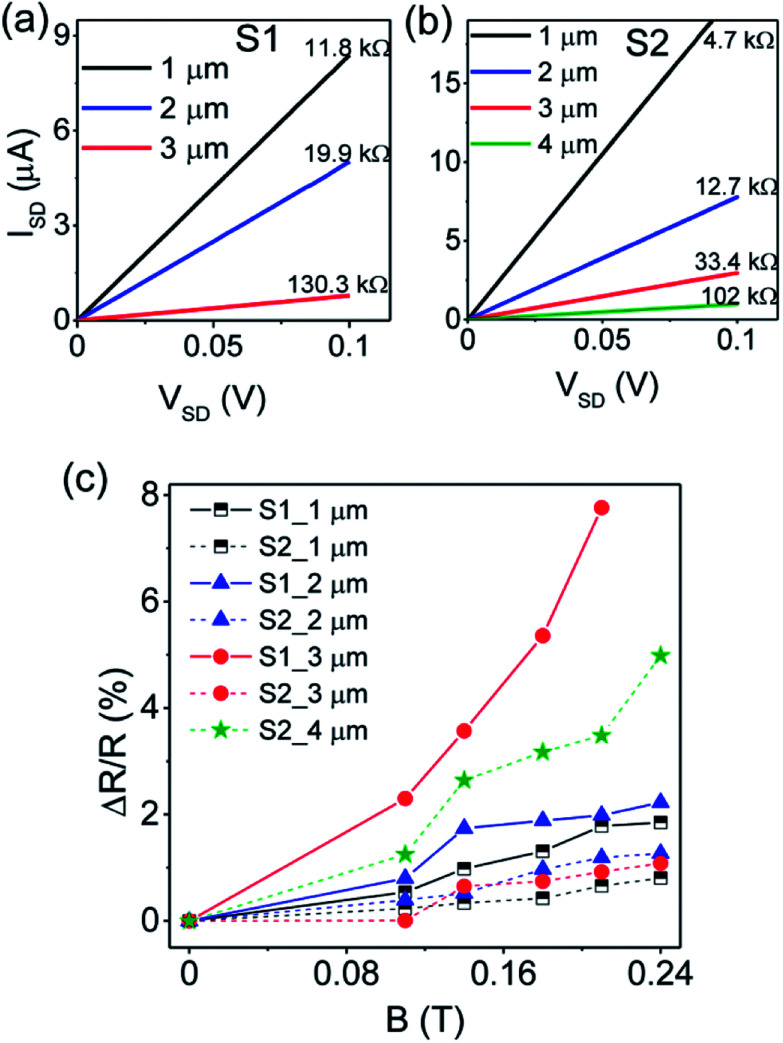
Characteristic *I*–*V* curves of SWNTs devices with different electrodes spacing for sample S1 (a) and sample S2 (b) at zero magnetic field. The obtained resistance is increasing with the increase of electrode spacing. (c) Magnetoresistance curves of SWNT devices with different electrodes spacing for sample S1 (solid lines) and sample S2 (dashed lines).

We explore the evolution of the obtained MR with the electrodes spacing. Fig. 3a shows *I*–*V* characteristics of the SWNTs devices with different electrodes spacing for the sample S1. In the absence of magnetic field, the obtained electrical resistance is 11.8 kΩ, 19.9 kΩ and 130.3 kΩ, for devices with electrode spacing of 1 μm, 2 μm and 3 μm, respectively. The *I*–*V* curves shown in Fig. 3b are for another sample S2 with zero-field resistance of 4.7 kΩ, 12.7 kΩ, 33.4 kΩ and 102 kΩ, for devices with electrode spacing of 1 μm, 2 μm, 3 μm and 4 μm, respectively. Linear current–voltage curves with low electrical resistances for both samples imply an ohmic contact between SWNTs and electrodes. It is very clear from both results that the resistance depends on the spacing between electrodes. The increase in electrode spacing results in the resistance increasing. This can be explained by the presence of the defects in SWNTs which contribute to a resistance proportional to the length of the nanotubes.^18^

The magnetic field dependence of MR for devices with different electrodes spacing is shown in Fig. 3c. As seen from the result, all devices from both samples S1 and S2 produced a positive MR. However, no direct dependence between the MR and the electrode spacing is observed. There is an overlap between the MR curves for almost all devices, which suggests that the MR is independent on the length of SWNTs.

As expected, our devices consists of a mixture of metallic and semiconducting SWNTs, which has always been a challenge for the fabrication of SWNTs based devices. The presence of one type of SWNTs will hinder the uniformity and the performance of the device for the other type. For instance, the presence of metallic SWNTs which carry high current will affect the switching behaviour and the performance of a field-effect transistor, causing significant degradation to the device. Therefore, it is important to explore the MR dependence with the metallic or semiconducting nature of SWNTs. Some effective growth methods to separate metallic to semiconducting SWNTs have been reported.^19,20^ Post-synthetic approaches have also been used such as current-induced electrical breakdown, which was proven to be effective owing to the high selectivity and complete removal of metallic SWNTs.^16,21–23^ Herein, we further investigate the change in MR behaviour as we gradually remove metallic SWNTs by electrical breakdown.

**Fig. 4:**
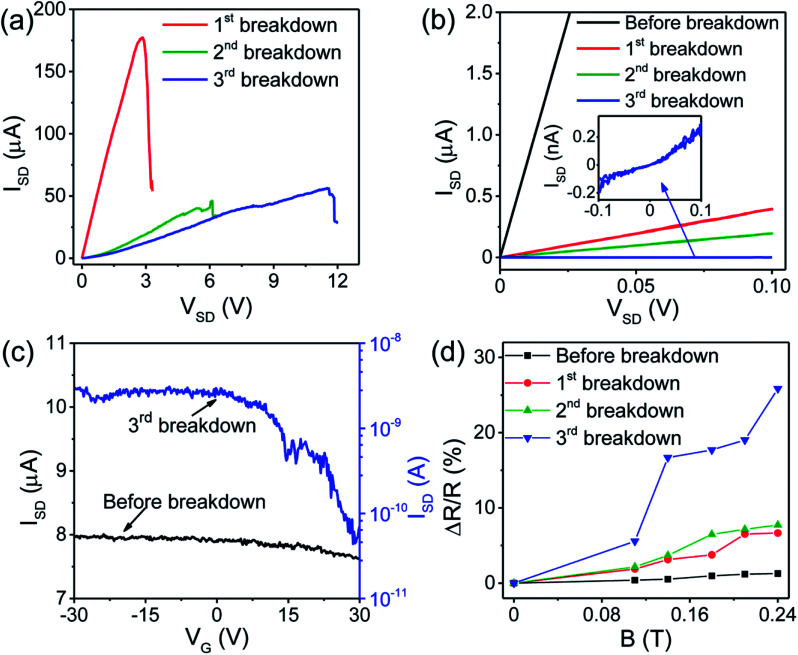
(a) Selective etching of metallic SWNTs by electrical breakdown at room temperature in air. (b) *I*–*V* curves before and after each cycle of breakdown. Inset shows the *I*–*V* curve after the third breakdown. There is no electrical hysteresis since same values of current are obtained in both forward and reverse direction of the *I*–*V* sweep. (c) Typical drain current *I*_SD_ (at bias *V*_SD_ = 100 mV) *versus* gate voltage *V*_G_ of the device at room temperature before breakdown (black line; left-hand scale) and after the third breakdown (blue line; right-hand scale). A large current modulation can be observed after the third breakdown (on/off ratio ∼10^2^), indicating a semiconducting nature of the remaining SWNTs. (d) Magnetic field dependence of magnetoresistance after each cycle of electrical breakdown.

Fig. 4a shows the *I*–*V* characteristics of the SWNT device during different cycles of electrical breakdown at room temperature. An increasing drain voltage (*V*_SD_) with a step of 100 μV was applied at the drain electrode. The drain current increases linearly with increasing voltage and suddenly drops which indicates the breakdown of some metallic SWNTs. The breakdown occurred at a voltage about 2.8 V during the first cycle of breakdown, about 6 V during the second cycle and reached about 11.6 V during the third breakdown. The increase in breakdown voltage indicates that the metallic to semiconducting ratio is gradually decreasing, which was confirmed by the gradual decrease in current after each breakdown cycle, as shown by *I*–*V* curves in Fig. 4b. The curves are linear before breakdown and after the first and second breakdown, while the third breakdown resulted in a non-linear curve as shown in the inset of Fig. 4b. No electrical hysteresis was found since the *I*–*V* sweep gives the same values of current in both forward and reverse direction. It should be noted that although the metallic SWNTs can be burned by the electrical breakdown, it is difficult to determine the ratio of metallic and semiconducting SWNTs by resistance change during electrical breakdown. For example, for the device shown in Fig. 4b, the original resistance is about 12.7 kΩ. After the first, second and third electrical breakdown, the resistance becomes 255 kΩ, 508 kΩ and 1 GΩ, respectively. The on/off current ratios are also analyzed. In Fig. 4c, typical drain current (*I*_SD_) *versus* gate voltage (*V*_G_) of the device at room temperature before breakdown and after the third breakdown is shown. Doped silicon was used as a back gate with 800 nm thick SiO_2_ insulating layer. A current flowing through SWNTs (*I*_SD_) is measured while sweeping the gate voltage (*V*_G_) between −30 V and +30 V at a fixed source-drain bias of 100 mV. A large current modulation can be observed after the third breakdown (on/off ratio ∼10^2^), indicating a semiconducting nature of the remaining SWNTs.

In Fig. 4d, we show the magnetic field dependence of MR after every cycle of breakdown. From the result, one can see that the magnitude of the MR increases with a gradual removal of metallic SWNTs. Since the original sample contains both semiconducting and metallic SWNTs, we imply that our MR results are the sum of MR contributions from metallic and semiconducting SWNTs. In addition, as all metallic SWNTs are completely removed during the third breakdown, one can deduce that the MR in semiconducting SWNTs is significantly larger than in metallic SWNTs. Before breakdown, a maximum MR value of about 1.5% at magnetic field of 0.24 tesla is observed. After the first and second breakdown, the MR increased to about 6% and 8%, respectively and becomes significantly larger (about 25%) for semiconducting SWNTs after the third breakdown. To validate our experimental results, we have measured the MR of another two devices, and the results are shown in the Fig. S2 and S3 in ESI.† Similar conclusions can be obtained that the MR of our SWNT devices can be enhanced after electrical breakdown, indicating the reproducibility of our results. The measurements were also done in both positive and negative magnetic fields (Fig. S3†) and the MR shows similar behaviour and almost the same magnitude in both positive and negative magnetic fields. In these devices, maximum values of MR about 20% (Fig. S2†) and 15% (Fig. S3†) were also obtained for semiconducting SWNTs. Meanwhile, the role of silver on the observed MR may be brought into question. However, based on the following reasons, the effect of silver is ruled out. Firstly, after annealing treatment, the silver aggregates on the top of Pd, which act as electrodes. Secondly, our electrical breakdown just etches away some SWNTs and the resulting MR should come from SWNTs rather than from the electrodes, which do not change before and after electrical breakdown.

Positive MR was previously reported in a mixture of metallic and semiconducting SWNT networks and films,^12,14^ and the obtained MR could be enhanced by lowering the temperature. A maximum MR value of about 10% was obtained by Kim *et al.* at temperatures *T* ≤ 3.8 K and magnetic field *B* ∼ 10 T.^12^ Meanwhile, Jhang *et al.* obtained the same MR value at *T* ∼ 1.5 K and *B* ∼ 30 T.^14^ The MR results obtained in our work are obviously higher since the measurements are done at room temperature and small magnetic fields (*B* ≤ 0.24 T). In addition, the obtained MR can be enhanced by selective removal of metallic SWNTs.

Other studies have reported the transport mechanism in semiconducting SWNTs.^13,24^ Suzuura *et al.* attributed the positive MR under perpendicular magnetic fields to the Anderson localization due to the random impurity scattering.^24^ Meanwhile, Yanagi *et al.* investigated the transport mechanism of SWNTs networks as a function of metallic to semiconducting ratio.^13^ They observed a crossover from weak localization to variable range hopping (VRH) as the metallic to semiconducting ratio decreased, attributing semiconducting SWNTs to be the source of localization. In our study, the MR is always positive and increases as the metallic SWNTs content is decreased. As for the mechanism of the MR effect in this work, it is attributed to the defect-induced magnetic moments in carbon nanotubes as reported previously.^25,26^ It should be noted that the exact reason for a larger MR effect in semiconducting SWNTs is still unclear. One possible reason is that the magnitude of magnetic moment at the defects depends somewhat on the metallic or semiconducting nature of SWNTs.

Semiconducting SWNTs are likely to play a key role in spintronic devices as their current could be controlled by both the gate and spin manipulation or nonvolatility. With the advances in a successful preparation of high purity semiconducting SWNTs, our study could prove useful for the development of high performance magnetoresistive devices that are operable at room temperature.

## Conclusions

In summary, we have studied the MR of SWNTs arrays at room temperature, under small magnetic fields up to 0.24 tesla. A positive MR was observed in a mixture of metallic and semiconducting SWNTs, which was independent on the length of the SWNTs between electrodes. Meanwhile, the magnitude of the obtained MR could be enhanced by selective removal of metallic SWNTs with electrical breakdown. Large positive MR values about 15%, 20% and 25% were found in three different devices at 0.24 tesla for semiconducting SWNTs at room temperature. We attribute the MR results to the defect-induced magnetic moments in carbon nanotubes. Even though the physical origin of the MR is not yet fully explored, our result suggests great potential of SWNTs in magneto-electronic devices.

## Conflicts of interest

There are no conflicts to declare.

## Supplementary Material

RA-008-C8RA00877A-s001
