# Supplementary material for: Large positive magnetoresistance in semiconducting single-walled carbon nanotubes at room temperature
Source: RSC Adv. 2018 Mar 13;8(19):10179–84. doi: 10.1039/c8ra00877a (PMC9078853; doi:10.1039/c8ra00877a)
Supplement: RA-008-C8RA00877A-s001 [file RA-008-C8RA00877A-s001.pdf]

Electronic Supplementary Material (ESI) for RSC Advances.
This journal is © The Royal Society of Chemistry 2018

Supplementary Information

Large Positive Magnetoresistance in Semiconducting Single-Walled Carbon Nanotubes at Room Temperature

Jean Pierre Nshimiyimana,[ab] Jian Zhang,[ab] Xiannian Chi,[ab] Xiao Hu,[ab] Pei Wu,[ab] Siyu Liu,[a] Jia Liu,[ab] Weiguo Chu*[a] and Lianfeng Sun*[a]

[a] *CAS Key Laboratory of Nanosystem and Hierarchical Fabrication, CAS Center for Excellence in Nanoscience, National Centre for Nanoscience and Technology, Beijing 100190, China.*

[b] *University of Chinese Academy of Sciences, Beijing 100049, China*

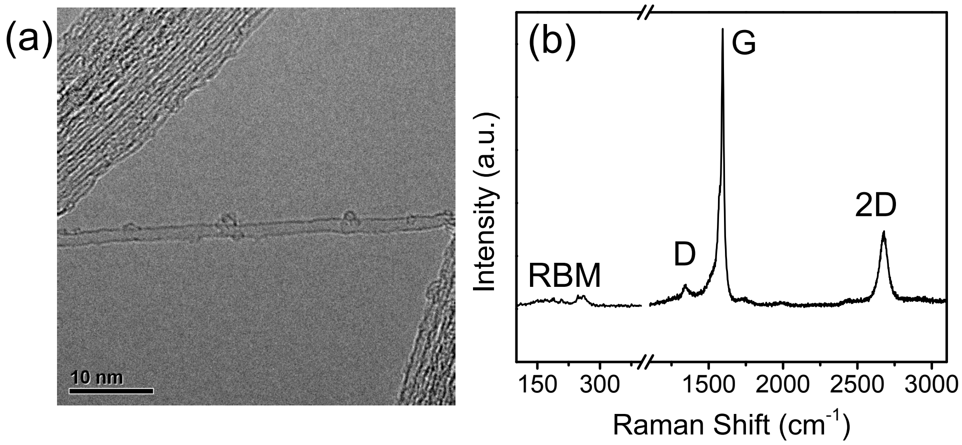

Fig. S1. Characterization of SWNTs (a) TEM and (b) Raman spectra of SWNTs used in this study.

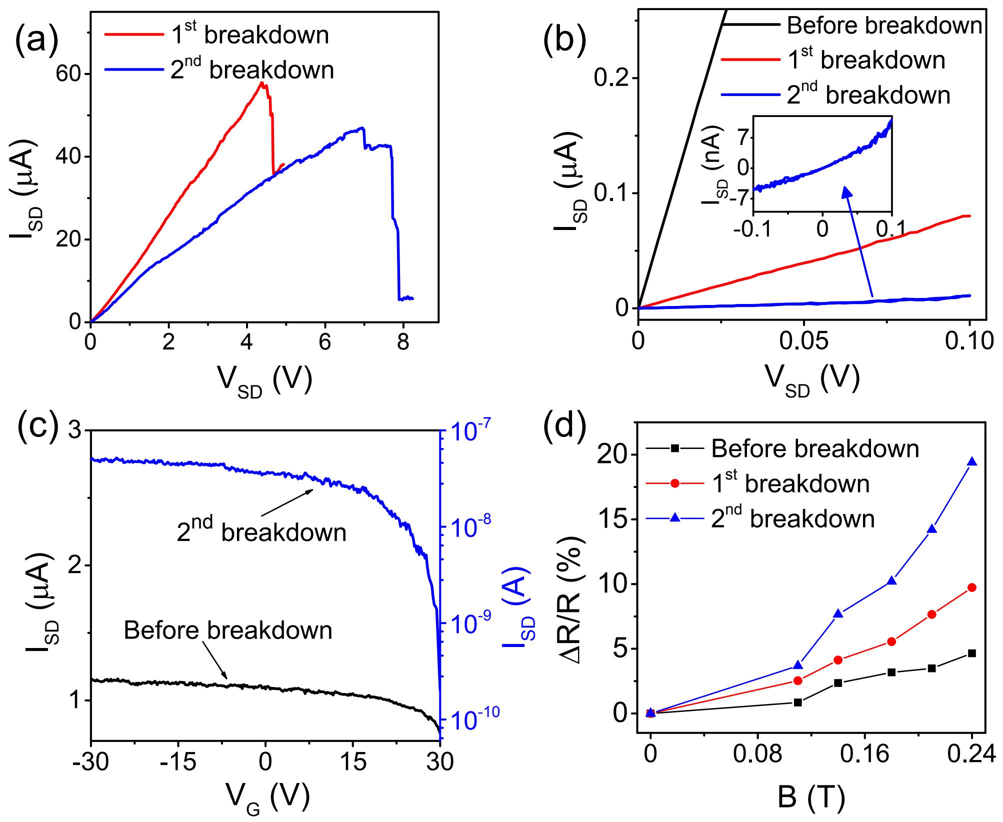

Fig. S2. MR measurements of the second SWNT device before and after electrical breakdown

(a) I-V characteristics during electrical breakdown at room temperature. (b) Characteristic I-V curves before and after each cycle of breakdown. Inset shows the I-V curve after the second breakdown. There is no electrical hysteresis since same values of current are obtained in both forward and reverse direction of the I-V sweep. (c) Typical drain current I_{SD} (at bias $V_{SD} = 100$ mV) versus gate voltage V_G of the device at room temperature before breakdown (black line; left-hand scale) and after the second breakdown (blue line; right-hand scale). (d) Magnetic field dependence of the MR after each cycle of electrical breakdown.

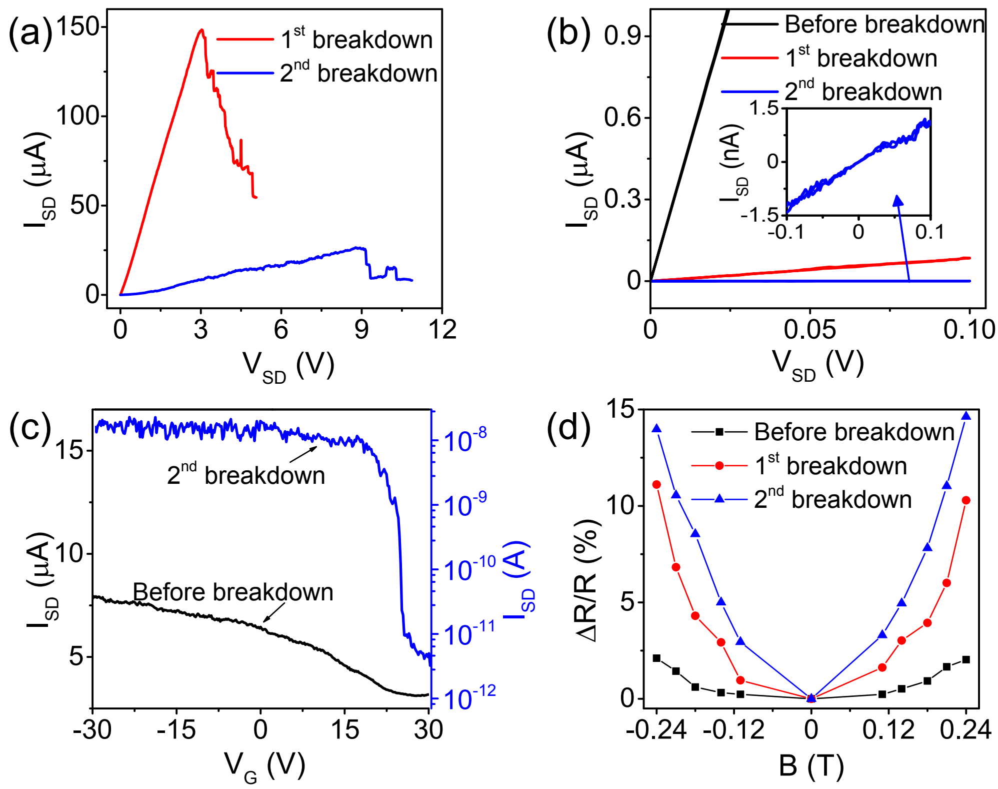

Fig. S3. MR measurements of the third SWNT device before and after electrical breakdown (a) I-V characteristics during electrical breakdown at room temperature. (b) Characteristic I-V curves before and after each cycle of breakdown. Inset is the I-V curve after the second breakdown which shows no electrical hysteresis. (c) Typical drain current I_{SD} (at bias V_{SD} = 100 mV) versus gate voltage V_G of the device at room temperature before breakdown (black line; left-hand scale) and after the second breakdown (blue line; right-hand scale). (d) Magnetic field dependence of the MR after each cycle of electrical breakdown. The MR shows similar behavior and almost the same magnitude in both positive and negative magnetic fields.
